# The Irreversible March of Time: Ischemic Delay and Impact on Outcomes in ST-Segment Elevation Myocardial Infarction

**DOI:** 10.3390/jcdd12120474

**Published:** 2025-12-02

**Authors:** Artur Dziewierz, Barbara Zdzierak, Wojciech Wańha, Giuseppe De Luca, Tomasz Rakowski

**Affiliations:** 1 2nd Department of Cardiology, Institute of Cardiology, Jagiellonian University Medical College, 30-688 Krakow, Poland; artur.dziewierz@uj.edu.pl (A.D.); barbarazdzierak@gmail.com (B.Z.); 2 Clinical Department of Cardiology and Cardiovascular Interventions, University Hospital, 30-688 Krakow, Poland; 3 Department of Cardiology and Structural Heart Diseases, Medical University of Silesia, 40-635 Katowice, Poland; wojciech.wanha@gmail.com; 4 Division of Cardiology, AOU Policlinico G Martino, University of Messina, 98124 Messina, Italy; giuseppe.deluca@uniume.it

**Keywords:** ST-segment elevation myocardial infarction, ischemic time, door-to-balloon time, reperfusion delay, occlusion myocardial infarction, STEMI networks

## Abstract

ST-segment elevation myocardial infarction (STEMI) represents a time-critical medical emergency where complete coronary artery occlusion initiates progressive myocardial necrosis. The fundamental principle of modern STEMI care—“Time is Muscle”—establishes that ischemic duration directly determines infarct size and clinical outcomes. Each minute of delay correlates with increased mortality, larger infarcts, and a higher risk of heart failure development. Total ischemic time encompasses both patient-mediated delays (often the largest component) and system-related delays, each influenced by distinct factors requiring targeted interventions. This comprehensive review analyzes the components of total ischemic time, quantifies the clinical consequences of delay, and evaluates evidence-based mitigation strategies. We examine the evolution from fibrinolysis to primary percutaneous coronary intervention and the resulting logistical challenges. System-level interventions—including public awareness campaigns, regionalized STEMI networks, pre-hospital ECG acquisition, and standardized hospital protocols—have dramatically reduced treatment times. However, persistent disparities based on geography, presentation timing, sex, race, and age remain problematic. Emerging technologies, particularly artificial intelligence for ECG interpretation, offer promise for further time reduction.

## 1. Introduction

### 1.1. The Emerging Paradigm: From STEMI to Occlusion Myocardial Infarction

**Figure 1:**
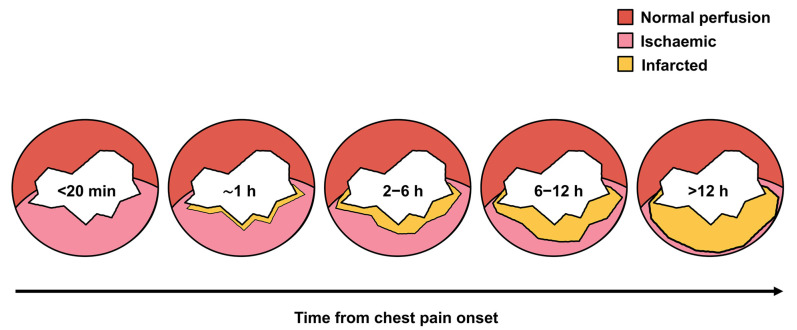
Time-dependent evolution of myocardial injury following coronary occlusion. Schematic representation of myocardial tissue viability over time following acute coronary occlusion in ST-segment elevation myocardial infarction. The diagram illustrates the progressive transition from normally perfused myocardium (red) through reversibly injured but salvageable tissue (pink, ischemic zone) to irreversibly damaged myocardium (yellow, infarcted zone). The area of salvageable tissue diminishes rapidly with increasing ischemic time, with the most dramatic losses occurring after 2–3 h. Early reperfusion therapy maximizes the myocardial salvage index by preserving the ischemic zone before irreversible injury occurs. The non-linear relationship between time and tissue loss underscores why the greatest clinical benefit occurs with reperfusion within the first 1–2 h after symptom onset.

ST-segment elevation myocardial infarction (STEMI) has traditionally been defined by complete, persistent occlusion of one or more coronary arteries, typically following atherosclerotic plaque rupture or erosion that triggers acute thrombosis [1,2,3]. This abrupt cessation of myocardial perfusion initiates ischemic injury progressing in a “wave-front” pattern from the vulnerable subendocardial layer outward toward the epicardium (Figure 1) [4,5]. While ST-segment elevation in two or more contiguous leads has been the hallmark diagnostic finding [1,2,3], emerging evidence reveals significant limitations of this paradigm.

For decades, the STEMI/non-ST-segment elevation MI (NSTEMI) dichotomy has guided acute MI management, relying on ST-segment elevation as the primary indicator for acute coronary occlusion requiring emergent reperfusion [1,2]. However, mounting evidence reveals critical limitations in this approach. Multiple large-scale meta-analyses involving tens of thousands of patients consistently demonstrate that approximately 25–33% of patients diagnosed with NSTEMI have a totally occluded culprit artery on subsequent angiography [6,7,8,9]. This misclassification has serious clinical implications. These patients face significantly higher risks of adverse outcomes, with a pooled short-term relative risk of all-cause mortality of 1.67 compared to NSTEMI patients without occlusion [10]. The excess mortality stems from systematic delays in reperfusion therapy: despite sharing the same underlying pathophysiology as STEMI patients, those with NSTEMI related to totally occluded culprit artery are denied emergent intervention due to the absence of classic ECG findings [7]. This recognition has prompted a paradigm shift toward the occlusion MI (OMI)/non-occlusion MI (NOMI) classification system [7,8,9]. The absence of classic ST-elevation in patients with a totally occluded culprit artery often results from well-developed collateral circulation. These collateral vessels can maintain sufficient myocardial viability to prevent the full-thickness transmural ischemia necessary to generate ST-segment elevation. The diagnosis incorporates multiple elements: classic ST-elevation, “STEMI-equivalent” patterns (De Winter T-waves, posterior MI patterns, hyperacute T-waves), refractory ischemic symptoms, and adjunctive tools like bedside echocardiography [8,9,11]. Also, when discussing inferior/posterior wall infarctions, it is crucial to emphasize that ST depressions often represent reciprocal changes mirroring ST elevations that would be visible with additional leads—particularly V7–V9 for posterior wall assessment. This highlights a critical limitation: while 12-lead ECG remains the standard, circumflex or marginal artery occlusions may manifest only as subtle depressions or remain entirely invisible without extended lead placement. This creates a dangerous paradox: while collateral circulation initially protects the myocardium, it masks the ECG signal that would trigger emergent reperfusion, leading directly to treatment delays associated with higher rates of cardiogenic shock and increased mortality in the NSTEMI with a totally occluded culprit artery population [7,12]. Additionally, the STEMI paradigm is compromised by a substantial false-positive rate, with 15–35% of emergency catheterization laboratory activations triggered by patients presenting with “STEMI mimics”. Conditions including pericarditis, benign early repolarization, and Takotsubo cardiomyopathy can produce ST-segment elevation that leads to unnecessary invasive procedures—interventions that are costly and potentially harmful to patients [7,13,14,15]. Despite compelling and growing evidence supporting the OMI paradigm, the newly released 2025 AHA/ACC Guideline for the Management of Patients With Acute Coronary Syndromes did not formally adopt the OMI/NOMI terminology, citing that foundational randomized clinical trials used the traditional STEMI/NSTEMI framework [1]. However, this stance is increasingly contrasted by international bodies; notably, the 2025 Australian Acute Coronary Syndromes guidelines have moved to adopt “Acute Coronary Occlusion MI” as a formal category, recognizing the necessity of treating the occlusion rather than the ECG trace alone. Proponents of the OMI paradigm argue that relying on historical trials perpetuates a “circular logic.” Critical examination of landmark fibrinolytic trials reveals they never angiographically differentiated between occlusive and non-occlusive MI, and many excluded patients without ST-elevation, potentially biasing the evidence base against this high-risk group [16]. Adherence to the STEMI paradigm based on this historical evidence may therefore perpetuate systematic under-treatment of patients with acute coronary occlusion who were misclassified within the very trials now cited as the gold standard [8]. Notably, the original investigators themselves cautioned against denying reperfusion therapy to patients without ST-elevation, noting that the data were insufficient to support such exclusion [17]. On the other hand, while this review uses “STEMI” terminology to reflect existing literature, we emphasize that the principles of timely reperfusion apply equally to all OMI patients—many of whom are currently missed by conventional approaches [7,8,9,18,19].

### 1.2. The “Time Is Muscle” Doctrine

Eugene Braunwald’s foundational concept that “Time is Muscle” underpins modern management of all acute coronary occlusions [20]. Final infarct size, directly proportional to ischemic duration, represents the primary predictor of long-term outcomes including heart failure development and mortality [20,21]. This relationship holds true whether patients present with classic STEMI or other OMI patterns [7]. The non-linear relationship between time and salvageable myocardium—with greatest benefit within the first hours—applies universally across the OMI spectrum (Figure 1). Importantly, infarct size is also shaped by the location of the occlusion (with anterior infarctions typically causing larger damage), extent of collateral circulation (better collaterals markedly reduce infarct size), and ischemic preconditioning (brief, controlled ischemia episodes locally or remotely can halve or significantly lessen myocardial necrosis) [20,21].

### 1.3. Evolution of Reperfusion Strategies

Despite therapeutic advances, in-hospital mortality in STEMI remains substantial at 4–12% in European registries [2,22,23,24]. Reperfusion therapy has evolved from pharmacological to mechanical approaches [1,2,25]. As detailed in Table 1, while fibrinolysis offers rapid deployment, its angiographic success rate (~65%) is significantly inferior to that of primary PCI (>95%). Consequently, primary percutaneous coronary intervention (PCI)—which mechanically opens the occluded vessel via balloon angioplasty, thrombectomy, and stenting—has become the preferred strategy, converting MI management from a pharmacological decision into a logistical challenge of rapid transport [2,26]. Its superior efficacy translates to lower rates of mortality, reinfarction, and stroke compared to fibrinolysis [27]. However, PCI’s effectiveness depends entirely on rapid access to specialized facilities and experienced teams [1,2,28]. Additionally, invasive strategies not only restore vessel patency but also visualize and stabilize the culprit lesion with stenting, making fibrinolysis—which cannot address the underlying plaque—no longer considered definitive treatment for MI.

The paradigm shift to primary PCI transformed acute coronary occlusion management from a pharmacological to a logistical challenge [28,29,30,31]. Additionally, reperfusion paradoxically induces further injury through oxidative stress and calcium overload, potentially accounting for up to 50% of final infarct size [32,33]. This underscores the absolute urgency of minimizing initial ischemic time before irreversible injury occurs—a principle that applies equally to all patients with acute coronary occlusion, whether meeting traditional STEMI criteria or presenting with other OMI patterns [1,2,21].

## 2. Components and Determinants of Total Ischemic Time

### 2.1. Patient-Mediated Delays

The interval from symptom onset to first medical contact (FMC) consistently represents the longest and most variable component of total ischemic time. Median patient decision delay approximates 100 min, constituting nearly 60% of pre-hospital delay [19,34,35,36].

**Figure 2:**
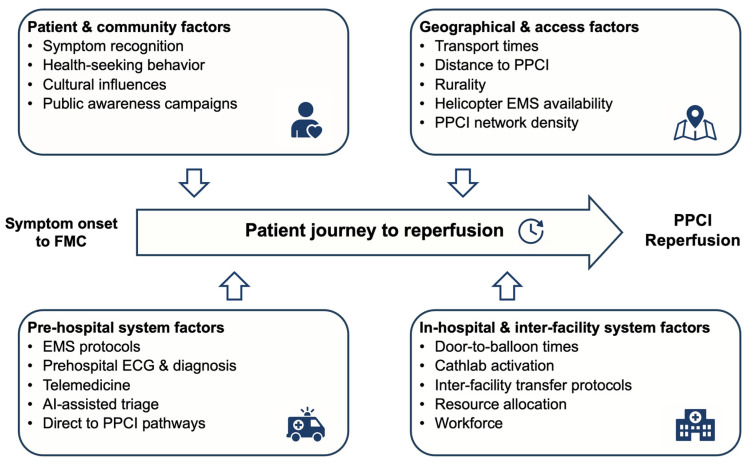
Factors affecting STEMI reperfusion timelines. This diagram shows the four key domains that influence time to reperfusion in STEMI patients: patient/community factors (symptom recognition, health-seeking behavior), geographical factors (transport times, distance to centers), pre-hospital system factors (EMS protocols, field diagnosis), and in-hospital factors (cathlab activation, transfer protocols). Targeted interventions within each domain may help reduce total ischemic time and improve clinical outcomes. Abbreviations: EMS = emergency medical services; FMC = first medical contact; PPCI = primary percutaneous coronary intervention; STEMI = ST-elevation myocardial infarction.

The major determinants of patient delay include (Table 2, Figure 2) [19,34,35,36,37,38,39,40,41]:

- Sociodemographic factors: advanced age, female sex, rural residence, low education, social isolation, diabetes mellitus
- Cognitive factors: symptom misinterpretation, particularly with atypical presentations (dyspnea, sweating, non-chest pain) common in women, elderly, and diabetics
- Behavioral factors: initial contact with general practitioners instead of emergency medical services (EMS) activation; self-transport versus ambulance utilization

Framing this as “patient delay” misleadingly assigns individual responsibility for what often reflects systemic public health failures. The American Heart Association recognizes patient delay as one of the “greatest obstacles” to successful STEMI care, calling for comprehensive public awareness campaigns [42].

### 2.2. Pre-Hospital System Delays

The pre-hospital phase from FMC/STEMI diagnosis to hospital arrival accounts for over 83% of symptom-to-treatment time [19,34,35,36,38,39,40,41]. Key components include:

- EMS response and scene time: guidelines recommend limiting scene time to <20 min, yet one-third of encounters exceed this benchmark, particularly in rural areas [43,44]
- Transport duration: influenced by distance, traffic, and weather conditions [43,44]
- Referral hospital delays (door-in-door-out, DIDO): for patients presenting to non-PCI-capable facilities, DIDO time represents a major delay source, occurring in 64% of transferred patients with median delays approaching one hour [43,44].

DIDO time emerges as a crucial determinant of overall treatment effectiveness. De Luca et al. provided evidence demonstrating that prolonged inter-hospital transfer delays were independently associated with impaired myocardial perfusion, increased infarct size, and significantly higher 1-year mortality rates in patients undergoing primary PCI [45].

### 2.3. In-Hospital System Delays

The in-hospital phase encompasses door-to-needle time for fibrinolysis and door-to-balloon time for primary PCI. Guidelines establish quality targets of door-to-needle ≤ 30 min and door-to-balloon ≤ 90 min [1,2]. The door-to-activation interval critically determines overall door-to-balloon time—achieving activation within 20 min yields 89% probability of meeting the 90 min goal versus 28% when exceeding 20 min [46]. Off-hours presentation, diagnostic uncertainty, and patient instability contribute to delays [47,48]. Table 2 summarizes the components and benchmarks of total ischemic time.

## 3. Clinical Consequences of Delayed Reperfusion

### 3.1. Mortality Impact

The relationship between reperfusion timing and mortality is both direct and continuous, yet its interpretation requires nuance. While shorter door-to-balloon times clearly benefit individual patients, population-level impacts have proven complex. Major registry data from the NCDR CathPCI Registry reveal a striking “door-to-balloon paradox”: despite significant improvements in national median door-to-balloon times (from 83 to 67 min), adjusted in-hospital mortality remained essentially unchanged at 4.7–5.0% [49]. This paradox likely stems from the fact that door-to-balloon time represents only a fraction of total ischemic time. Improvements in hospital metrics are easily negated if the pre-hospital phase—often the largest source of delay-remains unaddressed [1,2]. The focus must therefore shift to the entire chain of survival, from symptom onset to reperfusion. For individual patients, the risk associated with door-to-balloon time follows a continuous, non-linear trajectory. Landmark analyses demonstrate that adjusted in-hospital mortality rises progressively: from 3.0% at 30 min to 3.5% at 60 min, 4.3% at 90 min, and 7.0% at 150 min [50]. These data support an “as short as possible” reperfusion philosophy rather than a “meet the benchmark” approach, as every minute of delay increases risk [28,42,51,52]. 

However, excessive focus on the 90 min metric can produce unintended consequences. It may incentivize operators to choose faster femoral access over safer radial access or encourage selective exclusion of complex cases from quality reporting [1,2]. Furthermore, the mortality advantage of PCI over fibrinolysis diminishes with increasing PCI-related delay, reaching equipoise at 110–120 min [53,54,55]. For high-risk patients with large anterior infarctions presenting early, this threshold may be as short as 40–60 min [55,56].

### 3.2. Myocardial Salvage and Infarct Size

Delayed reperfusion directly increases final infarct size [5,20,57]. The myocardial salvage index—the proportion of area at risk preserved from necrosis—shows strong inverse correlation with ischemic time [58,59]. Reperfusion within two hours yields the greatest salvage and the highest myocardial salvage index [60]. Longer symptom-to-balloon times independently predict larger infarcts, increased microvascular obstruction, and reduced ejection fraction [58,59,61]. Higher myocardial salvage index powerfully predicts long-term event-free survival [60].

### 3.3. Long-Term Morbidity

Quantitatively, each 30 min reperfusion delay increases one-year mortality risk by 7.5% [62]. Larger infarcts from delayed reperfusion create substrate for adverse ventricular remodeling—progressive dilatation and dysfunction culminating in heart failure [63,64,65]. Patients presenting ≥12 h after symptom onset show significantly higher heart failure hospitalization rates: one-year death or heart failure hospitalization reaches 29% versus 17% for those treated within 12 h [66,67]. This reframes the speed imperative beyond preventing acute death to preventing chronic disease. While delayed reperfusion unequivocally worsens acute outcomes, the long-term prognostic differences between patients initially classified as STEMI versus NSTEMI have been debated [68]. Historically, NSTEMI has been associated with worse long-term prognosis, a finding often attributed to older age and higher comorbidity burden in this population. However, recent large-scale evidence from the 21,789-patient PRAISE registry challenges this paradigm [69]. After rigorous adjustment for baseline clinical and therapeutic variables, the study demonstrated that 1-year outcomes—including all-cause mortality, reinfarction, and major bleeding- were largely similar between STEMI and NSTEMI patients [69].

However, heart failure is not the only devastating long-term consequence. Extensive, transmural necrosis—a direct function of prolonged ischemic time—fundamentally compromises the structural and electrical integrity of the ventricle, leading to other life-threatening complications [70]. Mechanical complications arise in the acute to subacute phase (days to weeks post-MI) when necrotic, softened myocardium tears under systolic pressure. This can precipitate catastrophic events including ventricular septal defect, papillary muscle rupture with acute severe mitral regurgitation, or left ventricular free wall rupture, often resulting in cardiac tamponade and cardiogenic shock [71,72,73]. Chronically, non-contractile scar tissue can thin and bulge outward, forming a left ventricular aneurysm that impairs overall pump function and creates a region of relative stasis, thereby increasing the risk of thrombus formation and subsequent embolic stroke [72]. Electrical complications arise from the scar tissue that replaces necrotic myocardium [74]. This scar is electrically inert, and the border zone between scar and viable myocardium creates a heterogeneous substrate for reentrant electrical circuits. This architectural disruption forms the basis for increased long-term risk of life-threatening ventricular arrhythmias, including ventricular tachycardia and fibrillation, which can result in sudden cardiac death weeks, months, or even years after the initial MI. The risk of all these sequelae—progressive heart failure, mechanical rupture, and lethal arrhythmias—is directly proportional to final infarct size. Minimizing total ischemic time therefore represents the most powerful intervention available to prevent not only acute mortality but also lifelong cardiovascular morbidity [75].

### 3.4. Magnified Impact in High-Risk Populations

Delay’s impact is catastrophically amplified in cardiogenic shock patients, where mortality approaches 33–61% [76,77]. For every 10 min FMC-to-device delay between 60–90 min, absolute mortality increases 4–7% in shock patients versus <0.5% without shock [77]. This exponential relationship mandates risk-stratified triage protocols prioritizing the most unstable patients.

### 3.5. The Reperfusion Paradox

While minimizing total ischemic time remains paramount, restoring blood flow itself initiates myocardial ischemia-reperfusion injury (MIRI), a paradoxical process that can account for substantial final infarct size [78]. This “double-edged sword” of reperfusion has been a central challenge in MI care for decades [79]. Key mechanisms of MIRI include oxidative stress from reactive oxygen species, intracellular calcium overload leading to cardiomyocyte hypercontracture, sterile inflammation with neutrophil infiltration and microvascular dysfunction, and opening of the mitochondrial permeability transition pore (mPTP)—a critical step committing cells to death. Emerging evidence highlights NLRP3 inflammasome activation as a pivotal mechanism triggering pyroptosis, a highly pro-inflammatory form of programmed cell death that amplifies injury [78]. This understanding has fueled decades of investigation into adjunctive cardioprotective therapies, yet the field has been characterized by profound translational failure [80,81,82]. Strategies robustly effective in preclinical models consistently fail in large-scale human trials. Ischemic conditioning techniques—including remote ischemic conditioning (RIC) involving brief limb ischemia cycles—reliably reduce infarct size in animal models but showed no benefit in the large CONDI-2/ERIC-PPCI trial in STEMI patients [83,84]. Pharmacological approaches have fared no better. Cyclosporine, an mPTP inhibitor, demonstrated infarct size reduction in proof-of-concept studies but failed to improve clinical outcomes in the multicenter CIRCUS trial [85]. Sodium-hydrogen exchange inhibitors like cariporide failed in the GUARDIAN trial, and therapeutic hypothermia strategies, including selective intracoronary cooling (EURO-ICE trial), showed no benefit despite sound rationale [80,81,82,86,87]. This disconnect likely reflects fundamental disparities between simplified preclinical models using young, healthy animals and the complex reality of older STEMI patients with multiple comorbidities that alter cellular signaling. Routine comedications such as heparin may exert independent cardioprotective effects, further confounding results [78,79].

Despite these setbacks, several approaches show promise. Supersaturated oxygen (SSO_2_) therapy, involving intracoronary infusion of hyperbaric oxygen immediately post-PCI, reduces myocardial edema and improves microvascular flow. The AMIHOT and IC-HOT trials demonstrated significant infarct size reduction in anterior STEMI, leading to FDA approval in 2019—a rare translational success [88]. Mechanical unloading using transvalvular axial flow pumps (Impella CP) to reduce left ventricular work before reperfusion activates cardioprotective pathways and preserves mitochondrial integrity. The STEMI-DTU pilot trial proved this approach safe and feasible, with a pivotal trial ongoing [89]. However, the potential cardioprotective benefits must be carefully weighed against the additional ischemic time incurred during device insertion, underscoring the critical importance of operator experience and streamlined protocols to minimize procedural delay [78]. The STEMI-DTU pilot trial proved this approach safe and feasible, with a pivotal trial ongoing. Additional strategies under investigation include NLRP3 inflammasome inhibitors (colchicine, glyburide, novel specific inhibitors) and early intravenous metoprolol, which uniquely limits infarct size through direct neutrophil modulation as demonstrated in METOCARD-CNIC [78].

The future of cardioprotection will likely require multi-target approaches combining mechanical support, metabolic modulation, and targeted anti-inflammatory agents to address the multifaceted nature of MIRI [78,79]. While the search for effective adjunctive cardioprotection continues, the most potent ‘drug’ currently available remains the speed of reperfusion itself. Since we cannot yet pharmacologically neutralize the consequences of delay, we must rigorously optimize the logistical systems that deliver mechanical reperfusion.

## 4. Evidence-Based Strategies to Mitigate Delay

### 4.1. Public Health Initiatives

Given patient delay’s dominance, public awareness campaigns represent critical interventions [28,90]. One national campaign evaluation found 64% patient awareness, significantly associated with shorter patient delay (≤1 h) and pre-hospital delay (≤2 h) [91]. Effective campaigns address symptom misinterpretation, denial, and emphasize EMS activation over self-transport [1,2,91].

### 4.2. Optimizing Pre-Hospital Care

Pre-hospital strategies transform sequential care into parallel processing:

- Pre-hospital ECG: As a Class I recommendation, pre-hospital ECG serves as a cornerstone intervention in acute MI management [1,2]. A systematic review and meta-analysis demonstrated its association with substantial reductions in door-to-balloon time (mean difference > 26 min) and significantly lower short-term mortality (odds ratio 0.72) [92]. The survival benefit is most pronounced in high-risk subgroups, including patients with cardiogenic shock or diabetes [93]. This finding reframes pre-hospital ECG beyond its role as a time-saving tool—it becomes a critical instrument for early risk stratification, enabling healthcare systems to preferentially accelerate care for the most vulnerable patients [1,2]. The “Stent—Save a Life!” initiative recognizes pre-hospital ECG as fundamental to effective STEMI networks [28].
- Field catheterization lab activation: Reduces door-to-balloon by additional 15–45 min, enabling direct transport to catheterization lab bypassing emergency department [37,94,95,96]
- Regionalized networks: The “Stent—Save a Life!” initiative provides a structured methodology for establishing STEMI networks categorized by available resources: primary PCI networks (optimal), hub-and-spoke networks (acceptable long-term), pharmaco-invasive networks (transitional), and fibrinolysis networks (basic care requiring urgent upgrade) [1,2,28,94]. Direct transport protocols to PCI-capable centers significantly reduce mortality [96].

### 4.3. Streamlining In-Hospital Processes

Standardized “Code STEMI” protocols dramatically reduce door-to-balloon times [97,98,99]. Key components include:

- Emergency physician activation authority without cardiology consultation
- Single-call team notification systems
- 24/7 team availability within 20–30 min
- Regular performance feedback

National quality initiatives reduced median US door-to-balloon times from 94 min (2005) to <60 min currently [100]. The success of primary PCI extends beyond individual procedural excellence to require comprehensive system-wide organization with standardized operating procedures and rigorous time monitoring. Every component—from EMS activation and pre-hospital ECG transmission to catheterization lab mobilization and door-to-balloon times—must function as a coordinated chain with continuous quality metrics tracking. This systematic approach, with regular audits of time intervals and protocol adherence, transforms primary PCI from an isolated intervention into a high-reliability healthcare delivery system.

### 4.4. Fibrinolysis and Pharmaco-Invasive Strategy

When anticipated FMC-to-device time exceeds 120 min per ESC guidelines, the pharmaco-invasive strategy—early fibrinolysis followed by routine angiography within 2–24 h—provides a crucial alternative [1,2]. These recommendations are supported by results from a large network meta-analysis demonstrating that the pharmaco-invasive approach ranked second after primary PCI, with a mortality odds ratio of 0.79 (95% CI, 0.59–1.08) compared with conventional fibrinolytic therapy alone [101]. The “Stent—Save a Life!” initiative recognizes pharmaco-invasive networks as transitional solutions that should be upgraded to full PCI capability but acknowledges their critical role in providing timely reperfusion when geography or resources preclude immediate PCI access [28]. For patients facing unavoidable long delays, pharmaco-invasive strategy yields superior long-term survival compared to delayed primary PCI [31,102]. Healthcare systems must maintain flexible, hybrid approaches deploying appropriate reperfusion modality based on real-time assessment of geography and anticipated delay. However, implementing this flexibility requires maintaining pre-hospital fibrinolysis capability in ambulances, which poses significant practical challenges—drugs must be readily available, staff trained, and systems must achieve door-to-needle times within 10 min of STEMI recognition [1,2]. Given the sporadic indications for this strategy in most regions, healthcare systems face a fundamental choice between investing in rarely-used fibrinolysis infrastructure versus optimizing transfer networks to minimize delays to primary PCI [28].

### 4.5. Upstream Glycoprotein IIb-IIIa Inhibitors

Initial enthusiasm for this appealing strategy was supported by results from an individual patient data meta-analysis (EGYPT) [103,104], the On-TIME II trial pooled analysis [105,106], and several prospective registries [93,107,108]. These studies demonstrated benefits in pre- and post-procedural TIMI flow, reduced distal embolization, and improved survival with early versus late administration of glycoprotein (GP) IIb-IIIa inhibitors. However, the negative results of the FINESSE trial [109] substantially diminished interest in upstream GP IIb-IIIa inhibitor use, leading to its near abandonment and a Class III recommendation in clinical guidelines.

The FINESSE trial results [109] should be interpreted considering several limitations: relatively long ischemic times, potentially insufficient pretreatment duration (randomization was permitted at hub centers, thus including patients not requiring transfer), and a lower-risk patient profile compared to studies showing positive results. Indeed, subsequent subanalyses demonstrated clear benefits in high-risk patients who underwent transfer and had ischemic times < 4 h [110,111]. This observation aligns with the established relationship between thrombus composition and ischemic time, whereby platelets comprise a larger proportion of thrombi within the first three hours after symptom onset. The clinical relevance of time-dependent thrombus composition has been confirmed in subanalyses of both the large HORIZONS trial [112] and the On-TIME II study [113].

Zalunfiban, a novel subcutaneous GP IIb-IIIa inhibitor currently in development, may substantially improve STEMI treatment. This agent achieves rapid onset of action (≤15 min) following subcutaneous administration, with high-grade inhibition of platelet function in response to ADP and thrombin receptor agonists [114,115]. Several innovative features make zalunfiban an ideal candidate for upstream strategy in patients with acute coronary occlusion: user-friendly administration, short duration of action (~2 h) that may minimize bleeding risk, and reduced thrombocytopenia risk compared to current GP IIb-IIIa inhibitors due to its distinct mechanism of action [116]. The recent CELEBRATE trial [117,118] demonstrated that pre-hospital administration of zalunfiban significantly improved coronary patency and ST-segment resolution without increasing severe bleeding. These findings demonstrate that early antiplatelet therapy with zalunfiban may improve clinical outcomes in STEMI patients with an acceptable safety profile and this “pharmacological facilitation” may offer a bridge for patients with long transport times.

## 5. Persistent Challenges and Disparities

### 5.1. Geographic Disparities

Rural patients experience longer delays at every stage, receive less primary PCI, and more fibrinolysis [119]. Paradoxically, adjusted mortality shows no urban-rural difference [119], possibly reflecting higher baseline risk in urban populations receiving superior care that equalizes outcomes with lower-risk rural patients receiving inferior care. However, this pattern of rural disadvantage may not be universal. In contrast to these US findings, a recent French study found no difference in five-year outcomes in rural and urban groups [120]. This geographic variation suggests that rural-urban disparities in cardiac care may be significantly mitigated by local healthcare infrastructure and policies [121].

### 5.2. Temporal Disparities

The “weekend effect” persists in STEMI care, with off-hours presentation associated with longer door-to-balloon times and small but significant mortality increases [48,122,123]. Despite 24/7 protocols, equitable care regardless of arrival time remains unrealized [122]. These disparities may be further exacerbated during large-scale system stressors such as the COVID-19 pandemic [52,124,125,126] or regional armed conflicts [127,128]—which disrupt emergency networks, reallocate critical resources, and disproportionately amplify delays in already vulnerable off-hours or resource-limited settings.

### 5.3. Demographic Disparities

- Women: Women with STEMI consistently present at older ages with greater comorbidity burdens, including diabetes and hypertension, which complicate their clinical presentation [37,129,130,131,132,133,134]. They more frequently experience atypical symptoms—shortness of breath, nausea, fatigue, and interscapular pain—leading to diagnostic and care-seeking delays [37,131]. These factors result in less timely reperfusion therapy and higher rates of in-hospital complications, including stroke and major bleeding, ultimately contributing to increased mortality compared with men [1,2,135]. However, emerging evidence suggests these symptoms should not be labeled ‘atypical,’ but rather viewed as typical manifestations of distinct female-pattern ischemic heart disease [2]. Two key entities, both more prevalent in women, are central to understanding these sex-based differences. Myocardial infarction with non-obstructive coronary arteries (MINOCA) accounts for 5–10% of all MIs and is defined by evidence of MI without obstructive (≥50%) stenosis on angiography [2,136]. Women are disproportionately affected, comprising nearly 50% of the MINOCA population despite representing only approximately 25% of patients with MI from obstructive coronary disease. The mechanisms underlying MINOCA are heterogeneous and include plaque erosion (rather than rupture), coronary artery spasm, and spontaneous coronary artery dissection (SCAD)—all more common in women than men [137,138,139]. Additionally, coronary microvascular dysfunction (CMD) involves dysregulation of the myocardial microvasculature the smallest arteries and capillaries invisible on standard angiography. These microvessels fail to dilate appropriately, leading to genuine myocardial ischemia despite patent epicardial vessels. CMD is highly prevalent in women presenting with chest pain and non-obstructive coronary arteries and represents a primary mechanism underlying both MINOCA and chronic angina in this population [137,140]. This mechanistic framework reframes the diagnostic challenge. Classic crushing chest pain reflects acute occlusion of a large epicardial coronary artery [2]. The diffuse, patchy ischemia caused by CMD or transient occlusion from vasospasm logically generates a different symptom profile. These symptoms are typical for the underlying pathophysiology but are misclassified as “atypical” by a diagnostic paradigm constructed primarily from observations in male patients. This fundamental mismatch contributes directly to diagnostic delays, misclassification, and undertreatment that perpetuate worse outcomes in women [2,137].
- Racial/ethnic minorities: Black and Hispanic patients with STEMI face substantial disparities, experiencing lower odds of receiving timely, guideline-directed care such as prehospital ECGs and achieving door-to-balloon targets [141,142,143]. These populations consistently undergo invasive therapies like coronary angiography and PCI less frequently—a disparity that persists after adjusting for clinical and socioeconomic factors.
- Elderly: Older adults with STEMI experience particular vulnerability to systematic treatment delays, with the pre-hospital phase representing the most significant contributor [1,2,23,37,141,142,144]. These delays often stem from atypical presentations—confusion or weakness rather than chest pain—which patients and caregivers may attribute to other age-related conditions [145]. Even within established regionalized systems, elderly patients receive delayed reperfusion, partially explaining their elevated in-hospital mortality rates [1,2,145,146,147,148]. This pattern of undertreatment is substantiated by large-scale contemporary registry data. The PRAISE registry, for example, found that patients with NSTEMI—a group that disproportionately includes elderly and women- were significantly less likely to receive evidence-based secondary prevention medications at discharge compared to STEMI patients [69]. This contributes to a treatment-risk paradox, wherein patients with high comorbidity burden and significant long-term risk receive less intensive pharmacological therapy, further compounding the worse outcomes observed in elderly and women [69].

Notably, standardized protocols effectively eliminate these disparities [130,132]. Systems-based care approaches have demonstrated remarkable success in reducing inequities. One study showed that a comprehensive four-step protocol—incorporating ED catheterization laboratory activation, safe handoff checklists, immediate patient transfer, and radial-first PCI—successfully eliminated sex-based differences in door-to-balloon times and guideline-directed medical therapy administration [130]. This care standardization not only improved outcomes across all patient groups but also significantly narrowed the 30-day mortality gap between men and women, demonstrating that protocol-driven approaches represent powerful tools for achieving healthcare equity [130,132].

### 5.4. Challenges in Low- and Middle-Income Countries

**Figure 3:**
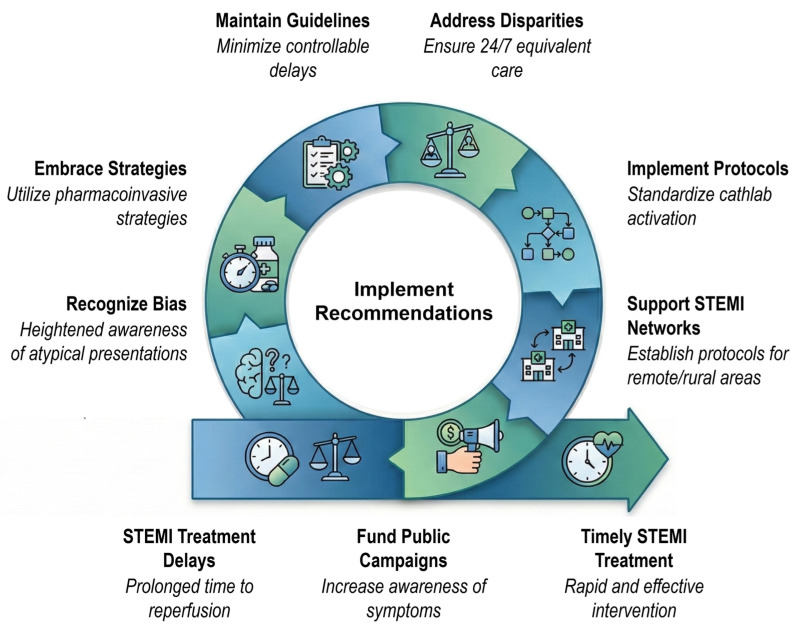
Strategies to reduce STEMI treatment delays. The diagram summarizes key system-level and patient-level interventions that collectively support timely reperfusion, including guideline adherence, standardized protocols, reduction in care disparities, network coordination, public education, and recognition of atypical presentations.

The challenges discussed thus far primarily reflect high-resource settings (Table 3). For the majority of the world’s population living in low- and middle-income countries, barriers to timely reperfusion are fundamentally different and more profound [54,149,150]. These healthcare systems face multiple interconnected challenges: inadequate or absent EMS, resulting in few ambulance arrivals; prolonged transit times (median 300 min to hospital presentation in India); severe shortages of PCI-capable facilities concentrated in urban centers; and critical deficits in trained specialists [150]. The most significant barrier, however, is the prohibitive out-of-pocket cost of primary PCI. This financial burden creates a “fear of finance” that both deters patients from seeking care and dictates treatment decisions [28,149,150]. Consequently, the pharmaco-invasive strategy—early fibrinolysis followed by planned PCI—represents not merely an alternative for managing long delays but often the only feasible reperfusion strategy for most of the population [2,28,150,151]. Recognizing these distinct realities is essential for developing globally relevant STEMI care strategies (Table 4, Figure 3).

## 6. Future Directions

### 6.1. Technological Innovation

- Artificial Intelligence: AI-ECG systems show promise for detecting not only classic STEMI but also subtle OMI patterns that traditional criteria often miss [8,9,152,153,154]. For instance, recent data on the “Queen of Hearts” AI model showed a sensitivity of 92% (vs. 71% standard care) and specificity of 81% (vs. 29%), drastically reducing false-positive cath lab activations [155]. However, AI implementation faces significant practical and ethical barriers [156]. Practical challenges include high development costs, requirements for vast quantities of high-quality, unbiased training data, and the technical complexity of integrating AI tools with fragmented hospital IT systems [157]. Ethical and social challenges prove equally profound. Algorithmic bias may cause models to underperform in populations underrepresented in training data. Automation complacency risks clinicians over-relying on AI suggestions, while selective adherence may lead them to follow only recommendations that confirm pre-existing beliefs [157]. The “black box” problem of AI transparency and the need for clear accountability frameworks for AI-driven decisions must be addressed before widespread adoption [158]. Progress requires rigorous evaluation and cautious, ethically grounded implementation—not merely technological advancement.
- Telemedicine: Real-time communication platforms between field crews and PCI centers reduce diagnostic uncertainty and optimize preparation [2,17,28]. Fifth-generation cellular technology provides the critical infrastructure for advanced mobile healthcare, offering robust communication pipeline which transforms ambulances into mobile diagnostic hubs, enabling high-definition video consultations and seamless transmission of large data files from paramedic-performed ultrasounds [159]. The technology allows expert-level clinical decision-making to begin at the patient’s bedside [160].
- Wearable-based MI prediction: While consumer smartwatches and other wearables demonstrate efficacy in detecting arrhythmias such as atrial fibrillation, their application for acute MI diagnosis remains unvalidated and confronts substantial technical limitations, including inadequate signal quality and the absence of 12-lead ECG equivalency [161]. In the immediate future, these devices will likely serve primarily in long-term cardiovascular risk stratification and preliminary abnormality detection for subsequent clinical evaluation, rather than functioning as primary diagnostic instruments for acute STEMI [158,161].
- Re-evaluating prehospital pharmacotherapy: Routine prehospital administration of P2Y_12_ inhibitors (“pretreatment”) has been largely discontinued following disappointing trial results [1,2]. Current 2024 ESC and 2025 AHA/ACC guidelines reflect this clinical shift by recommending dual antiplatelet therapy without mandating prehospital initiation [1,2]. On the other hand, the landscape may evolve with the introduction of subcutaneous glycoprotein IIb-IIIa inhibitors. Another, possibility might be administration of agents mitigating ischemia/reperfusion injury [162,163]. Future research will likely emphasize selective, individualized approaches rather than universal pretreatment protocols.

### 6.2. System Evolution

Future STEMI care requires fully integrated regional “chains of survival” functioning as coordinated units from 1-1-2/9-1-1 call to reperfusion [1,2,164]. The “Stent—Save a Life!” initiative outlines a systematic approach for network development, defining implementation phases [28]:

- Preparation: Establish task force and action plan with regional stakeholders
- Mapping: Identify PCI/non-PCI centers, assess transport times, confirm EMS availability
- Building: Assign roles based on available resources and network type
- Quality Assessment: Monitor key performance indicators continuously

Essential network characteristics include 24/7 service availability, structured cooperation following standardized protocols, regular stakeholder meetings, and continuous self-assessment [2,17,28]. Sustaining performance demands transparent auditing and feedback on metrics including presentation timing, treatment rates, procedural success, and mortality.

### 6.3. Research Priorities

Critical knowledge gaps include [2,17]:

- Optimal timing for pharmaco-invasive PCI (2–24 h window) and new subcutaneous upstream antithrombotic therapies;
- OMI/NOMI versus STEMI/NSTEMI triage trials;
- Effective public awareness campaign design;
- Targeted interventions for persistent disparities;
- Prospective validation of AI technologies;
- Multi-target cardioprotection strategies in high-risk populations.

## 7. Conclusions

A foundational principle of modern cardiology, supported by overwhelming evidence, is that every minute of coronary artery occlusion results in quantifiable and irreversible myocardial loss. Every minute from symptom onset to reperfusion increases mortality, infarct size, and heart failure risk—whether patients present with classic STEMI or other OMI patterns. The emerging OMI paradigm reveals that 25–34% of patients with acute coronary occlusion are missed by traditional STEMI criteria, experiencing systematic treatment delays despite similar pathophysiology and outcomes. While system-based approaches have achieved remarkable improvements through regional networks, pre-hospital protocols, and standardized hospital processes, significant challenges persist. These challenges encompass variable patient delays, failure to recognize the full spectrum of acute coronary occlusion, and profound care disparities driven by geography, demographics, and socioeconomic factors—particularly in low- and middle-income countries. AI-based ECG interpretation capable of detecting the complete range of OMI patterns offers a promising pathway to ensure timely reperfusion for all patients with acute coronary occlusion. The battle against time in STEMI requires coordinated, evidence-based, equitable care extending from patient’s home to catheterization laboratory. By targeting each delay source with proven interventions (Table 4), the medical community can continue improving survival and preserving quality of life for patients experiencing this devastating emergency.

## Figures and Tables

**Table 1:** Comparison of reperfusion strategies.

Feature	Primary PCI	Fibrinolysis	Pharmaco-Invasive
Mechanism	Mechanical opening	Thrombus dissolution	Initial lysis followed by coronary angiogram/PCI
Time indication	FMC-to-device ≤ 120 min	PCI unavailable or >120 min	FMC-to-device > 120 min
Advantages	>95% success definitive treatment	Rapid deployment anywhere	Combines speed with definitive therapy
Disadvantages	Time-dependent; infrastructure needs	~65% success bleeding risk	Intracranial hemorrhage risk requires coordination
Delayed presentation efficacy	Benefit diminishes significantly	Efficacy declines after hours	Superior to delayed primary PCI

Abbreviations: FMC = first medical contact; PCI = percutaneous coronary intervention.

**Table 2:** Components and benchmarks of total ischemic time in STEMI.

Time Interval	Definition	Guideline Target	Common Delay Sources
Patient delay	Symptom onset to FMC	Minimize	Symptom misinterpretation, denial, general practitioner contact, self-transport
Pre-hospital system	FMC to hospital arrival	Minimize	EMS dispatch, scene time, transport distance
Door-in-door-out	Non-PCI hospital arrival to departure	≤30 min	Transport availability, ED processes, diagnostics
FMC/Door-to-ECG	FMC/Hospital arrival to ECG	≤10 min	Triage delays, symptom recognition failure
Door-to-activation	Hospital arrival to cath lab activation	≤20 min	ECG interpretation, decision-making
FMC/Door-to-balloon	FMC/Hospital arrival to device inflation	≤90 min	Team assembly, complex procedures, instability
Total ischemic time	Symptom onset to reperfusion	≤120 min (optimal)	All combined delays

Abbreviations: FMC = first medical contact; EMS = emergency medical services; ED = emergency department; ECG = electrocardiogram; PCI = percutaneous coronary intervention.

**Table 3:** Comparative barriers to timely reperfusion in high- and low-resource settings.

Barrier Domain	High-Resource Setting	Low/Middle-Income Setting
Patient/community	Symptom misinterpretation; denial; failure to use EMS	Lack of basic awareness; fear of catastrophic cost; reliance on traditional medicine
Pre-hospital system	EMS on-scene time; inter-hospital transfer delays; “weekend effect”	Lack of organized EMS; no pre-hospital ECG/ triage; long transport over poor infrastructure
In-hospital system	Cath lab activation delays; ED dwell time; simultaneous presentations	Paucity of PCI-capable centers; lack of trained specialists; inability to provide 24/7 service
Financial	Insurance co-pays/deductibles; market share competition between hospitals	Prohibitive out-of-pocket cost of PCI; lack of universal health coverage
Primary reperfusion strategy	Primary PCI (default)	Pharmaco-invasive (often the only feasible option)

Abbreviations: EMS = emergency medical services; ED = emergency department; ECG = electrocardiogram; PCI = percutaneous coronary intervention.

**Table 4:** Targeted interventions to minimize STEMI treatment delays.

Stakeholder	Key Recommendations	Specific Actions
Policymakers & public health officials	Fund sustained public awareness campaigns	Focus on typical and atypical symptoms Emphasize immediate activation of emergency medical services (e.g., 1-1-2/9-1-1) Ensure cultural competency
	Support regional STEMI networks	Define protocols for rural/remote areas Ensure pharmaco-invasive strategy availability Mandate performance reporting
Healthcare system leaders	Implement standardized protocols	Establish emergency physician activation authority Deploy single-call notification systems Monitor performance continuously
	Address temporal disparities	Ensure 24/7 equivalent care quality Optimize off-hours staffing models
	Use standardization to promote equity	Reduce care variability Target vulnerable populations
Clinicians	Maintain guideline adherence	Minimize all controllable delays Focus on door-to-ECG and door-to-balloon metrics
	Embrace flexible strategies	Utilize pharmaco-invasive approach when appropriate Implement risk-stratified triage protocols
	Recognize bias potential	Maintain heightened awareness for atypical presentations Address disparities proactively
Researchers	Identify priority research areas	Develop patient delay reduction strategies Create disparity elimination interventions Optimize pharmaco-invasive approaches Validate AI-based risk stratification and triage tools in diverse populations

Abbreviations: AI = artificial intelligence; ECG = electrocardiogram; STEMI = ST-elevation myocardial infarction.

## Abbreviations

The following abbreviations are used in this manuscript:

ACC	American College of Cardiology
AHA	American Heart Association
AI	Artificial intelligence
ED	Emergency department
ECG	Electrocardiogram
EMS	Emergency medical services
ESC	European Society of Cardiology
FMC	First medical contact
GP	Glycoprotein
MI	Myocardial infarction
NOMI	Non-occlusion myocardial infarction
NSTEMI	Non-ST-segment elevation myocardial infarction
OMI	Occlusion myocardial infarction
PCI	Percutaneous coronary intervention
PPCI	Primary percutaneous coronary intervention
SCAI	Society for Cardiovascular Angiography and Interventions
STEMI	ST-segment elevation myocardial infarction
TIMI	Thrombolysis In Myocardial Infarction (flow grade)
